# Using KOOS-PS to validate dichotomous global ratings of improvement or worsening following total knee arthroplasty

**DOI:** 10.2340/17453674.2024.42632

**Published:** 2024-12-23

**Authors:** Daniel L RIDDLE, Levent DUMENCI

**Affiliations:** 1 Physical Therapy, Orthopaedic Surgery and Rheumatology, Virginia Commonwealth University, Richmond, VA; 2 College of Public Health, Department of Epidemiology and Biostatistics Temple University, Philadelphia, PA, USA

*Sir*,—The study by Winther and colleagues used KOOS-PS scores as the gold standard to judge the diagnostic potential for 1-year global ratings of change (i.e.. ratings of “better,” “same,” “unable to discriminate,” or “worse,” dichotomized to either worse or all remaining ratings) to judge outcome 1 year following total knee arthroplasty (TKA) [1]. The authors used a classic diagnostic test design. They determined whether the dichotomized global rating could “diagnose” good versus poor outcome as determined by preoperative to 1-year KOOS-PS change scores, dichotomized to either worse (negative change) or no change or better (positive change) over a 1-year postoperative period.

We have written about and studied sources of error for both patient-reported outcome measures (PROMs) such as KOOS and global ratings of change following TKA [2,3]. Many sources of error exist for both PROMS and global ratings [2,3]. KOOS-PS scores, for example, have less than perfect reliability, error is enhanced by deriving difference scores relative to individual scores, and dichotomizing difference score results in substantial loss of information and also adds error. Global ratings are vulnerable to recall bias, the 4 nominal categories add error and both validity and reliability are further compromised by the “worse” versus “all other” dichotomization. These issues were not discussed in the paper.

The investigators asked patients to use the global rating scale to rate the extent of change in function 1 year following surgery compared with the preoperative functional status. Lingard used a substantially shorter timeframe of 3 months post-TKA to judge patient recall of preoperative functional status items and found poor to fair agreement (i.e., weighted kappa, 0.20–0.41) [4]. We conducted a Kappa analysis of the extent of chance-corrected agreement between the dichotomized global rating and the dichotomized KOOS-PS score of improved versus worsened reported by Winther et al. and found a Kappa of 0.28, 95% confidence interval 0.22–0.34, a very similar Kappa to that reported by Lingard et al. [4] and that falls squarely in the fair range [5]. The reason for the low chance-corrected agreement despite the high observed agreement of 89% reported by Winther et al. [1] is attributable to the highly unbalanced data distribution. While 89% of the observations agreed, the percentage agreement expected by chance alone was 84%. We note that Winther and colleagues [1] did not address reliability of the KOOS-PS, the global ratings, or the extent of chance corrected agreement for the 2 measures. Winther and colleagues also did not address the fallibility of the KOOS-PS measure, which, for the reasons cited above, is not an acceptable gold standard for judging meaningful change following TKA.

Given the error in the gold standard, and the several important sources of error for both measures, we call into question the conclusion made by the authors that “high agreement between the anchor question and the KOOS-PS demonstrated that the KOOS-PS can be replaced with an anchor question to assess change in function after primary TKA.”
